# Supplementary material for: Pyruvate Dehydrogenase Kinase 1 inhibition mediated oxidative phosphorylation enhancement in cartilage promotes osteoarthritis progression
Source: BMC Musculoskelet Disord. 2023 Jul 20;24:597. doi: 10.1186/s12891-023-06585-6 (PMC10357736; doi:10.1186/s12891-023-06585-6)
Supplement: Supplementary file 1 — Additional file 1. [file 12891_2023_6585_MOESM1_ESM.zip › MF.Gsea.1653627316242/GOMF_GLYCOSYLTRANSFERASE_ACTIVITY.html]

Details for gene set GOMF\_GLYCOSYLTRANSFERASE\_ACTIVITY[GSEA]

|  || Dataset | 55457\_collapsed\_to\_symbols.cls.cls #OA\_versus\_normal.cls.cls #OA\_versus\_normal\_repos |
| Phenotype | cls.cls#OA\_versus\_normal\_repos |
| Upregulated in class | OA |
| GeneSet | GOMF\_GLYCOSYLTRANSFERASE\_ACTIVITY |
| Enrichment Score (ES) | 0.2271303 |
| Normalized Enrichment Score (NES) | 1.1656772 |
| Nominal p-value | 0.12341772 |
| FDR q-value | 0.60516906 |
| FWER p-Value | 1.0 |
Table: GSEA Results Summary

  

Fig 1: Enrichment plot: GOMF\_GLYCOSYLTRANSFERASE\_ACTIVITY      
 Profile of the Running ES Score & Positions of GeneSet Members on the Rank Ordered List

  

| SYMBOL | TITLE | RANK IN GENE LIST | RANK METRIC SCORE | RUNNING ES | CORE ENRICHMENT || 1 | C1GALT1C1 | C1GALT1 specific chaperone 1 [Source:HGNC Symbol;Acc:HGNC:24338] | 22 | 1.098 | 0.0161 | Yes |
| 2 | B3GALNT1 | "beta-1,3-N-acetylgalactosaminyltransferase 1 (globoside blood group) [Source:HGNC Symbol;Acc:HGNC:918]" | 101 | 0.914 | 0.0248 | Yes |
| 3 | ST8SIA1 | "ST8 alpha-N-acetyl-neuraminide alpha-2,8-sialyltransferase 1 [Source:HGNC Symbol;Acc:HGNC:10869]" | 133 | 0.873 | 0.0366 | Yes |
| 4 | GALNT11 | polypeptide N-acetylgalactosaminyltransferase 11 [Source:HGNC Symbol;Acc:HGNC:19875] | 164 | 0.845 | 0.0479 | Yes |
| 5 | EXT2 | exostosin glycosyltransferase 2 [Source:HGNC Symbol;Acc:HGNC:3513] | 170 | 0.838 | 0.0612 | Yes |
| 6 | GLT8D2 | glycosyltransferase 8 domain containing 2 [Source:HGNC Symbol;Acc:HGNC:24890] | 181 | 0.830 | 0.0739 | Yes |
| 7 | GLT8D1 | glycosyltransferase 8 domain containing 1 [Source:HGNC Symbol;Acc:HGNC:24870] | 221 | 0.804 | 0.0839 | Yes |
| 8 | MGAT4C | MGAT4 family member C [Source:HGNC Symbol;Acc:HGNC:30871] | 234 | 0.796 | 0.0958 | Yes |
| 9 | B4GAT1 | "beta-1,4-glucuronyltransferase 1 [Source:HGNC Symbol;Acc:HGNC:15685]" | 282 | 0.764 | 0.1046 | Yes |
| 10 | EXTL2 | exostosin like glycosyltransferase 2 [Source:HGNC Symbol;Acc:HGNC:3516] | 357 | 0.722 | 0.1105 | Yes |
| 11 | PIGP | phosphatidylinositol glycan anchor biosynthesis class P [Source:HGNC Symbol;Acc:HGNC:3046] | 367 | 0.718 | 0.1214 | Yes |
| 12 | UGT2B4 | UDP glucuronosyltransferase family 2 member B4 [Source:HGNC Symbol;Acc:HGNC:12553] | 566 | 0.634 | 0.1162 | Yes |
| 13 | MGAT4A | "alpha-1,3-mannosyl-glycoprotein 4-beta-N-acetylglucosaminyltransferase A [Source:HGNC Symbol;Acc:HGNC:7047]" | 657 | 0.604 | 0.1189 | Yes |
| 14 | ALG6 | "ALG6 alpha-1,3-glucosyltransferase [Source:HGNC Symbol;Acc:HGNC:23157]" | 696 | 0.595 | 0.1256 | Yes |
| 15 | PARP2 | poly(ADP-ribose) polymerase 2 [Source:HGNC Symbol;Acc:HGNC:272] | 758 | 0.579 | 0.1302 | Yes |
| 16 | ALG8 | "ALG8 alpha-1,3-glucosyltransferase [Source:HGNC Symbol;Acc:HGNC:23161]" | 900 | 0.543 | 0.1279 | Yes |
| 17 | ALG5 | ALG5 dolichyl-phosphate beta-glucosyltransferase [Source:HGNC Symbol;Acc:HGNC:20266] | 945 | 0.529 | 0.1330 | Yes |
| 18 | B3GALT4 | "beta-1,3-galactosyltransferase 4 [Source:HGNC Symbol;Acc:HGNC:919]" | 962 | 0.526 | 0.1403 | Yes |
| 19 | HYAL1 | hyaluronidase 1 [Source:HGNC Symbol;Acc:HGNC:5320] | 997 | 0.518 | 0.1461 | Yes |
| 20 | PIGV | phosphatidylinositol glycan anchor biosynthesis class V [Source:HGNC Symbol;Acc:HGNC:26031] | 1064 | 0.503 | 0.1490 | Yes |
| 21 | C1GALT1 | "core 1 synthase, glycoprotein-N-acetylgalactosamine 3-beta-galactosyltransferase 1 [Source:HGNC Symbol;Acc:HGNC:24337]" | 1110 | 0.494 | 0.1535 | Yes |
| 22 | GALNT10 | polypeptide N-acetylgalactosaminyltransferase 10 [Source:HGNC Symbol;Acc:HGNC:19873] | 1173 | 0.481 | 0.1565 | Yes |
| 23 | APRT | adenine phosphoribosyltransferase [Source:HGNC Symbol;Acc:HGNC:626] | 1178 | 0.481 | 0.1640 | Yes |
| 24 | GYG1 | glycogenin 1 [Source:HGNC Symbol;Acc:HGNC:4699] | 1209 | 0.474 | 0.1693 | Yes |
| 25 | RPN2 | ribophorin II [Source:HGNC Symbol;Acc:HGNC:10382] | 1294 | 0.459 | 0.1702 | Yes |
| 26 | B4GALT7 | "beta-1,4-galactosyltransferase 7 [Source:HGNC Symbol;Acc:HGNC:930]" | 1411 | 0.442 | 0.1682 | Yes |
| 27 | B3GNT2 | "UDP-GlcNAc:betaGal beta-1,3-N-acetylglucosaminyltransferase 2 [Source:HGNC Symbol;Acc:HGNC:15629]" | 1427 | 0.439 | 0.1742 | Yes |
| 28 | HEXB | hexosaminidase subunit beta [Source:HGNC Symbol;Acc:HGNC:4879] | 1434 | 0.438 | 0.1808 | Yes |
| 29 | ALG9 | "ALG9 alpha-1,2-mannosyltransferase [Source:HGNC Symbol;Acc:HGNC:15672]" | 1487 | 0.431 | 0.1837 | Yes |
| 30 | ABO | "ABO, alpha 1-3-N-acetylgalactosaminyltransferase and alpha 1-3-galactosyltransferase [Source:HGNC Symbol;Acc:HGNC:79]" | 1495 | 0.429 | 0.1902 | Yes |
| 31 | B4GALT6 | "beta-1,4-galactosyltransferase 6 [Source:HGNC Symbol;Acc:HGNC:929]" | 1500 | 0.429 | 0.1968 | Yes |
| 32 | FUT8 | fucosyltransferase 8 [Source:HGNC Symbol;Acc:HGNC:4019] | 1565 | 0.418 | 0.1986 | Yes |
| 33 | GCNT3 | "glucosaminyl (N-acetyl) transferase 3, mucin type [Source:HGNC Symbol;Acc:HGNC:4205]" | 1683 | 0.401 | 0.1959 | Yes |
| 34 | SIRT5 | sirtuin 5 [Source:HGNC Symbol;Acc:HGNC:14933] | 1758 | 0.390 | 0.1964 | Yes |
| 35 | ST8SIA4 | "ST8 alpha-N-acetyl-neuraminide alpha-2,8-sialyltransferase 4 [Source:HGNC Symbol;Acc:HGNC:10871]" | 1850 | 0.379 | 0.1954 | Yes |
| 36 | XYLT2 | xylosyltransferase 2 [Source:HGNC Symbol;Acc:HGNC:15517] | 1938 | 0.368 | 0.1945 | Yes |
| 37 | PYGM | "glycogen phosphorylase, muscle associated [Source:HGNC Symbol;Acc:HGNC:9726]" | 1952 | 0.367 | 0.1995 | Yes |
| 38 | PARP16 | poly(ADP-ribose) polymerase family member 16 [Source:HGNC Symbol;Acc:HGNC:26040] | 1997 | 0.361 | 0.2019 | Yes |
| 39 | GCNT1 | glucosaminyl (N-acetyl) transferase 1 [Source:HGNC Symbol;Acc:HGNC:4203] | 2035 | 0.357 | 0.2048 | Yes |
| 40 | UGT2B17 | UDP glucuronosyltransferase family 2 member B17 [Source:HGNC Symbol;Acc:HGNC:12547] | 2050 | 0.356 | 0.2095 | Yes |
| 41 | QPRT | quinolinate phosphoribosyltransferase [Source:HGNC Symbol;Acc:HGNC:9755] | 2077 | 0.353 | 0.2132 | Yes |
| 42 | ART3 | ADP-ribosyltransferase 3 (inactive) [Source:HGNC Symbol;Acc:HGNC:725] | 2101 | 0.349 | 0.2170 | Yes |
| 43 | CHPF2 | chondroitin polymerizing factor 2 [Source:HGNC Symbol;Acc:HGNC:29270] | 2281 | 0.329 | 0.2083 | Yes |
| 44 | FUT4 | fucosyltransferase 4 [Source:HGNC Symbol;Acc:HGNC:4015] | 2318 | 0.324 | 0.2107 | Yes |
| 45 | FUT7 | fucosyltransferase 7 [Source:HGNC Symbol;Acc:HGNC:4018] | 2343 | 0.322 | 0.2141 | Yes |
| 46 | PYGB | glycogen phosphorylase B [Source:HGNC Symbol;Acc:HGNC:9723] | 2400 | 0.315 | 0.2148 | Yes |
| 47 | CSGALNACT1 | chondroitin sulfate N-acetylgalactosaminyltransferase 1 [Source:HGNC Symbol;Acc:HGNC:24290] | 2507 | 0.302 | 0.2113 | Yes |
| 48 | PIGH | phosphatidylinositol glycan anchor biosynthesis class H [Source:HGNC Symbol;Acc:HGNC:8964] | 2573 | 0.294 | 0.2110 | Yes |
| 49 | ST6GALNAC5 | "ST6 N-acetylgalactosaminide alpha-2,6-sialyltransferase 5 [Source:HGNC Symbol;Acc:HGNC:19342]" | 2641 | 0.286 | 0.2104 | Yes |
| 50 | RFNG | RFNG O-fucosylpeptide 3-beta-N-acetylglucosaminyltransferase [Source:HGNC Symbol;Acc:HGNC:9974] | 2646 | 0.286 | 0.2147 | Yes |
| 51 | PIGB | phosphatidylinositol glycan anchor biosynthesis class B [Source:HGNC Symbol;Acc:HGNC:8959] | 2663 | 0.284 | 0.2181 | Yes |
| 52 | UGT2A3 | UDP glucuronosyltransferase family 2 member A3 [Source:HGNC Symbol;Acc:HGNC:28528] | 2854 | 0.265 | 0.2074 | Yes |
| 53 | B3GALT2 | "beta-1,3-galactosyltransferase 2 [Source:HGNC Symbol;Acc:HGNC:917]" | 2883 | 0.262 | 0.2095 | Yes |
| 54 | UMPS | uridine monophosphate synthetase [Source:HGNC Symbol;Acc:HGNC:12563] | 2936 | 0.257 | 0.2096 | Yes |
| 55 | GALNT7 | polypeptide N-acetylgalactosaminyltransferase 7 [Source:HGNC Symbol;Acc:HGNC:4129] | 2954 | 0.255 | 0.2124 | Yes |
| 56 | B3GAT3 | "beta-1,3-glucuronyltransferase 3 [Source:HGNC Symbol;Acc:HGNC:923]" | 2987 | 0.251 | 0.2139 | Yes |
| 57 | HEXA | hexosaminidase subunit alpha [Source:HGNC Symbol;Acc:HGNC:4878] | 3025 | 0.247 | 0.2150 | Yes |
| 58 | PLOD1 | "procollagen-lysine,2-oxoglutarate 5-dioxygenase 1 [Source:HGNC Symbol;Acc:HGNC:9081]" | 3155 | 0.235 | 0.2087 | Yes |
| 59 | POGLUT2 | protein O-glucosyltransferase 2 [Source:HGNC Symbol;Acc:HGNC:19350] | 3238 | 0.227 | 0.2059 | Yes |
| 60 | GTDC1 | glycosyltransferase like domain containing 1 [Source:HGNC Symbol;Acc:HGNC:20887] | 3273 | 0.223 | 0.2069 | Yes |
| 61 | UGT1A8 | UDP glucuronosyltransferase family 1 member A8 [Source:HGNC Symbol;Acc:HGNC:12540] | 3301 | 0.221 | 0.2083 | Yes |
| 62 | EXT1 | exostosin glycosyltransferase 1 [Source:HGNC Symbol;Acc:HGNC:3512] | 3321 | 0.219 | 0.2104 | Yes |
| 63 | MTAP | methylthioadenosine phosphorylase [Source:HGNC Symbol;Acc:HGNC:7413] | 3324 | 0.219 | 0.2138 | Yes |
| 64 | GBA | glucosylceramidase beta [Source:HGNC Symbol;Acc:HGNC:4177] | 3327 | 0.219 | 0.2172 | Yes |
| 65 | ST3GAL6 | "ST3 beta-galactoside alpha-2,3-sialyltransferase 6 [Source:HGNC Symbol;Acc:HGNC:18080]" | 3339 | 0.218 | 0.2199 | Yes |
| 66 | B4GALT4 | "beta-1,4-galactosyltransferase 4 [Source:HGNC Symbol;Acc:HGNC:927]" | 3407 | 0.211 | 0.2180 | Yes |
| 67 | PARP1 | poly(ADP-ribose) polymerase 1 [Source:HGNC Symbol;Acc:HGNC:270] | 3410 | 0.210 | 0.2213 | Yes |
| 68 | MFNG | MFNG O-fucosylpeptide 3-beta-N-acetylglucosaminyltransferase [Source:HGNC Symbol;Acc:HGNC:7038] | 3442 | 0.207 | 0.2222 | Yes |
| 69 | ST6GAL1 | "ST6 beta-galactoside alpha-2,6-sialyltransferase 1 [Source:HGNC Symbol;Acc:HGNC:10860]" | 3502 | 0.202 | 0.2208 | Yes |
| 70 | AGL | "amylo-alpha-1, 6-glucosidase, 4-alpha-glucanotransferase [Source:HGNC Symbol;Acc:HGNC:321]" | 3537 | 0.199 | 0.2214 | Yes |
| 71 | TYMP | thymidine phosphorylase [Source:HGNC Symbol;Acc:HGNC:3148] | 3588 | 0.195 | 0.2206 | Yes |
| 72 | PLOD3 | "procollagen-lysine,2-oxoglutarate 5-dioxygenase 3 [Source:HGNC Symbol;Acc:HGNC:9083]" | 3614 | 0.192 | 0.2218 | Yes |
| 73 | TNKS | tankyrase [Source:HGNC Symbol;Acc:HGNC:11941] | 3622 | 0.192 | 0.2244 | Yes |
| 74 | POMGNT1 | "protein O-linked mannose N-acetylglucosaminyltransferase 1 (beta 1,2-) [Source:HGNC Symbol;Acc:HGNC:19139]" | 3641 | 0.191 | 0.2260 | Yes |
| 75 | ST3GAL5 | "ST3 beta-galactoside alpha-2,3-sialyltransferase 5 [Source:HGNC Symbol;Acc:HGNC:10872]" | 3667 | 0.188 | 0.2271 | Yes |
| 76 | ALG12 | "ALG12 alpha-1,6-mannosyltransferase [Source:HGNC Symbol;Acc:HGNC:19358]" | 3723 | 0.183 | 0.2258 | No |
| 77 | PYGL | glycogen phosphorylase L [Source:HGNC Symbol;Acc:HGNC:9725] | 3826 | 0.174 | 0.2206 | No |
| 78 | GCNT4 | glucosaminyl (N-acetyl) transferase 4 [Source:HGNC Symbol;Acc:HGNC:17973] | 3858 | 0.169 | 0.2209 | No |
| 79 | DPY19L1 | dpy-19 like C-mannosyltransferase 1 [Source:HGNC Symbol;Acc:HGNC:22205] | 3875 | 0.167 | 0.2224 | No |
| 80 | XYLT1 | xylosyltransferase 1 [Source:HGNC Symbol;Acc:HGNC:15516] | 3916 | 0.163 | 0.2219 | No |
| 81 | ZC3HAV1 | "zinc finger CCCH-type containing, antiviral 1 [Source:HGNC Symbol;Acc:HGNC:23721]" | 3946 | 0.161 | 0.2222 | No |
| 82 | SIRT3 | sirtuin 3 [Source:HGNC Symbol;Acc:HGNC:14931] | 4009 | 0.155 | 0.2198 | No |
| 83 | ALG3 | "ALG3 alpha-1,3- mannosyltransferase [Source:HGNC Symbol;Acc:HGNC:23056]" | 4096 | 0.146 | 0.2154 | No |
| 84 | ST8SIA2 | "ST8 alpha-N-acetyl-neuraminide alpha-2,8-sialyltransferase 2 [Source:HGNC Symbol;Acc:HGNC:10870]" | 4153 | 0.142 | 0.2133 | No |
| 85 | SIRT4 | sirtuin 4 [Source:HGNC Symbol;Acc:HGNC:14932] | 4477 | 0.115 | 0.1898 | No |
| 86 | STT3A | STT3 oligosaccharyltransferase complex catalytic subunit A [Source:HGNC Symbol;Acc:HGNC:6172] | 4618 | 0.103 | 0.1805 | No |
| 87 | COLGALT2 | collagen beta(1-O)galactosyltransferase 2 [Source:HGNC Symbol;Acc:HGNC:16790] | 4703 | 0.096 | 0.1754 | No |
| 88 | B3GAT1 | "beta-1,3-glucuronyltransferase 1 [Source:HGNC Symbol;Acc:HGNC:921]" | 4715 | 0.095 | 0.1761 | No |
| 89 | PIGQ | phosphatidylinositol glycan anchor biosynthesis class Q [Source:HGNC Symbol;Acc:HGNC:14135] | 4825 | 0.085 | 0.1689 | No |
| 90 | UGT1A10 | UDP glucuronosyltransferase family 1 member A10 [Source:HGNC Symbol;Acc:HGNC:12531] | 4850 | 0.083 | 0.1684 | No |
| 91 | POFUT1 | protein O-fucosyltransferase 1 [Source:HGNC Symbol;Acc:HGNC:14988] | 4910 | 0.079 | 0.1650 | No |
| 92 | MGAT5 | "alpha-1,6-mannosylglycoprotein 6-beta-N-acetylglucosaminyltransferase [Source:HGNC Symbol;Acc:HGNC:7049]" | 5086 | 0.066 | 0.1523 | No |
| 93 | B3GALT5 | "beta-1,3-galactosyltransferase 5 [Source:HGNC Symbol;Acc:HGNC:920]" | 5132 | 0.061 | 0.1498 | No |
| 94 | DPY19L4 | dpy-19 like 4 [Source:HGNC Symbol;Acc:HGNC:27829] | 5133 | 0.061 | 0.1508 | No |
| 95 | MGAT2 | "alpha-1,6-mannosyl-glycoprotein 2-beta-N-acetylglucosaminyltransferase [Source:HGNC Symbol;Acc:HGNC:7045]" | 5317 | 0.046 | 0.1371 | No |
| 96 | MGAT1 | "alpha-1,3-mannosyl-glycoprotein 2-beta-N-acetylglucosaminyltransferase [Source:HGNC Symbol;Acc:HGNC:7044]" | 5407 | 0.040 | 0.1308 | No |
| 97 | PARP4 | poly(ADP-ribose) polymerase family member 4 [Source:HGNC Symbol;Acc:HGNC:271] | 5568 | 0.027 | 0.1186 | No |
| 98 | GALNT4 | polypeptide N-acetylgalactosaminyltransferase 4 [Source:HGNC Symbol;Acc:HGNC:4126] | 5610 | 0.023 | 0.1158 | No |
| 99 | GYG2 | glycogenin 2 [Source:HGNC Symbol;Acc:HGNC:4700] | 5756 | 0.011 | 0.1045 | No |
| 100 | B3GALT1 | "beta-1,3-galactosyltransferase 1 [Source:HGNC Symbol;Acc:HGNC:916]" | 5864 | 0.004 | 0.0962 | No |
| 101 | UGT8 | UDP glycosyltransferase 8 [Source:HGNC Symbol;Acc:HGNC:12555] | 5908 | 0.002 | 0.0928 | No |
| 102 | GALNT6 | polypeptide N-acetylgalactosaminyltransferase 6 [Source:HGNC Symbol;Acc:HGNC:4128] | 5915 | 0.001 | 0.0924 | No |
| 103 | MGAT4B | "alpha-1,3-mannosyl-glycoprotein 4-beta-N-acetylglucosaminyltransferase B [Source:HGNC Symbol;Acc:HGNC:7048]" | 5956 | -0.002 | 0.0893 | No |
| 104 | RXYLT1 | ribitol xylosyltransferase 1 [Source:HGNC Symbol;Acc:HGNC:13530] | 6030 | -0.007 | 0.0836 | No |
| 105 | LFNG | LFNG O-fucosylpeptide 3-beta-N-acetylglucosaminyltransferase [Source:HGNC Symbol;Acc:HGNC:6560] | 6161 | -0.017 | 0.0737 | No |
| 106 | EXTL1 | exostosin like glycosyltransferase 1 [Source:HGNC Symbol;Acc:HGNC:3515] | 6193 | -0.020 | 0.0716 | No |
| 107 | GALNT1 | polypeptide N-acetylgalactosaminyltransferase 1 [Source:HGNC Symbol;Acc:HGNC:4123] | 6217 | -0.022 | 0.0701 | No |
| 108 | SIRT2 | sirtuin 2 [Source:HGNC Symbol;Acc:HGNC:10886] | 6279 | -0.026 | 0.0657 | No |
| 109 | GYS2 | glycogen synthase 2 [Source:HGNC Symbol;Acc:HGNC:4707] | 6316 | -0.029 | 0.0634 | No |
| 110 | COLGALT1 | collagen beta(1-O)galactosyltransferase 1 [Source:HGNC Symbol;Acc:HGNC:26182] | 6535 | -0.045 | 0.0470 | No |
| 111 | ST8SIA5 | "ST8 alpha-N-acetyl-neuraminide alpha-2,8-sialyltransferase 5 [Source:HGNC Symbol;Acc:HGNC:17827]" | 6565 | -0.047 | 0.0454 | No |
| 112 | RPN1 | ribophorin I [Source:HGNC Symbol;Acc:HGNC:10381] | 6618 | -0.051 | 0.0422 | No |
| 113 | POMT1 | protein O-mannosyltransferase 1 [Source:HGNC Symbol;Acc:HGNC:9202] | 6652 | -0.053 | 0.0404 | No |
| 114 | GALNT14 | polypeptide N-acetylgalactosaminyltransferase 14 [Source:HGNC Symbol;Acc:HGNC:22946] | 6762 | -0.060 | 0.0329 | No |
| 115 | HPRT1 | hypoxanthine phosphoribosyltransferase 1 [Source:HGNC Symbol;Acc:HGNC:5157] | 6954 | -0.076 | 0.0191 | No |
| 116 | LALBA | lactalbumin alpha [Source:HGNC Symbol;Acc:HGNC:6480] | 7143 | -0.092 | 0.0058 | No |
| 117 | LARGE1 | LARGE xylosyl- and glucuronyltransferase 1 [Source:HGNC Symbol;Acc:HGNC:6511] | 7164 | -0.093 | 0.0057 | No |
| 118 | UGT2B11 | UDP glucuronosyltransferase family 2 member B11 [Source:HGNC Symbol;Acc:HGNC:12545] | 7270 | -0.100 | -0.0009 | No |
| 119 | GYS1 | glycogen synthase 1 [Source:HGNC Symbol;Acc:HGNC:4706] | 7345 | -0.105 | -0.0050 | No |
| 120 | PARP8 | poly(ADP-ribose) polymerase family member 8 [Source:HGNC Symbol;Acc:HGNC:26124] | 7404 | -0.111 | -0.0078 | No |
| 121 | DPAGT1 | dolichyl-phosphate N-acetylglucosaminephosphotransferase 1 [Source:HGNC Symbol;Acc:HGNC:2995] | 7452 | -0.115 | -0.0096 | No |
| 122 | GALNT2 | polypeptide N-acetylgalactosaminyltransferase 2 [Source:HGNC Symbol;Acc:HGNC:4124] | 7453 | -0.115 | -0.0078 | No |
| 123 | PARP12 | poly(ADP-ribose) polymerase family member 12 [Source:HGNC Symbol;Acc:HGNC:21919] | 7536 | -0.121 | -0.0122 | No |
| 124 | DPM1 | "dolichyl-phosphate mannosyltransferase subunit 1, catalytic [Source:HGNC Symbol;Acc:HGNC:3005]" | 7723 | -0.135 | -0.0247 | No |
| 125 | B4GALNT1 | "beta-1,4-N-acetyl-galactosaminyltransferase 1 [Source:HGNC Symbol;Acc:HGNC:4117]" | 7799 | -0.141 | -0.0283 | No |
| 126 | CHPF | chondroitin polymerizing factor [Source:HGNC Symbol;Acc:HGNC:24291] | 7960 | -0.152 | -0.0384 | No |
| 127 | GALNT8 | polypeptide N-acetylgalactosaminyltransferase 8 [Source:HGNC Symbol;Acc:HGNC:4130] | 8124 | -0.164 | -0.0485 | No |
| 128 | B3GNT3 | "UDP-GlcNAc:betaGal beta-1,3-N-acetylglucosaminyltransferase 3 [Source:HGNC Symbol;Acc:HGNC:13528]" | 8361 | -0.183 | -0.0641 | No |
| 129 | SIRT6 | sirtuin 6 [Source:HGNC Symbol;Acc:HGNC:14934] | 8532 | -0.196 | -0.0743 | No |
| 130 | UGGT2 | UDP-glucose glycoprotein glucosyltransferase 2 [Source:HGNC Symbol;Acc:HGNC:15664] | 8568 | -0.199 | -0.0738 | No |
| 131 | GCNT2 | glucosaminyl (N-acetyl) transferase 2 (I blood group) [Source:HGNC Symbol;Acc:HGNC:4204] | 8619 | -0.202 | -0.0745 | No |
| 132 | QTRT1 | queuine tRNA-ribosyltransferase catalytic subunit 1 [Source:HGNC Symbol;Acc:HGNC:23797] | 8620 | -0.202 | -0.0712 | No |
| 133 | ART1 | ADP-ribosyltransferase 1 [Source:HGNC Symbol;Acc:HGNC:723] | 8719 | -0.210 | -0.0755 | No |
| 134 | ST6GALNAC2 | "ST6 N-acetylgalactosaminide alpha-2,6-sialyltransferase 2 [Source:HGNC Symbol;Acc:HGNC:10867]" | 8870 | -0.222 | -0.0837 | No |
| 135 | FUT5 | fucosyltransferase 5 [Source:HGNC Symbol;Acc:HGNC:4016] | 8898 | -0.225 | -0.0822 | No |
| 136 | MGAT3 | "beta-1,4-mannosyl-glycoprotein 4-beta-N-acetylglucosaminyltransferase [Source:HGNC Symbol;Acc:HGNC:7046]" | 8920 | -0.226 | -0.0801 | No |
| 137 | SIRT1 | sirtuin 1 [Source:HGNC Symbol;Acc:HGNC:14929] | 8947 | -0.227 | -0.0785 | No |
| 138 | PIGZ | phosphatidylinositol glycan anchor biosynthesis class Z [Source:HGNC Symbol;Acc:HGNC:30596] | 9047 | -0.235 | -0.0825 | No |
| 139 | B4GALT5 | "beta-1,4-galactosyltransferase 5 [Source:HGNC Symbol;Acc:HGNC:928]" | 9051 | -0.236 | -0.0789 | No |
| 140 | PARP3 | poly(ADP-ribose) polymerase family member 3 [Source:HGNC Symbol;Acc:HGNC:273] | 9186 | -0.246 | -0.0854 | No |
| 141 | POMT2 | protein O-mannosyltransferase 2 [Source:HGNC Symbol;Acc:HGNC:19743] | 9216 | -0.249 | -0.0836 | No |
| 142 | PGAP4 | post-GPI attachment to proteins GalNAc transferase 4 [Source:HGNC Symbol;Acc:HGNC:28180] | 9347 | -0.259 | -0.0897 | No |
| 143 | FUT9 | fucosyltransferase 9 [Source:HGNC Symbol;Acc:HGNC:4020] | 9433 | -0.268 | -0.0920 | No |
| 144 | ALG13 | ALG13 UDP-N-acetylglucosaminyltransferase subunit [Source:HGNC Symbol;Acc:HGNC:30881] | 9444 | -0.268 | -0.0884 | No |
| 145 | UGT2A1 | UDP glucuronosyltransferase family 2 member A1 complex locus [Source:HGNC Symbol;Acc:HGNC:12542] | 9451 | -0.269 | -0.0845 | No |
| 146 | B3GNT4 | "UDP-GlcNAc:betaGal beta-1,3-N-acetylglucosaminyltransferase 4 [Source:HGNC Symbol;Acc:HGNC:15683]" | 9500 | -0.274 | -0.0838 | No |
| 147 | UGGT1 | UDP-glucose glycoprotein glucosyltransferase 1 [Source:HGNC Symbol;Acc:HGNC:15663] | 9516 | -0.275 | -0.0805 | No |
| 148 | PIGA | phosphatidylinositol glycan anchor biosynthesis class A [Source:HGNC Symbol;Acc:HGNC:8957] | 9571 | -0.280 | -0.0803 | No |
| 149 | PNP | purine nucleoside phosphorylase [Source:HGNC Symbol;Acc:HGNC:7892] | 9657 | -0.288 | -0.0823 | No |
| 150 | A4GALT | "alpha 1,4-galactosyltransferase (P blood group) [Source:HGNC Symbol;Acc:HGNC:18149]" | 9766 | -0.297 | -0.0859 | No |
| 151 | CSGALNACT2 | chondroitin sulfate N-acetylgalactosaminyltransferase 2 [Source:HGNC Symbol;Acc:HGNC:24292] | 9773 | -0.298 | -0.0815 | No |
| 152 | POGLUT1 | protein O-glucosyltransferase 1 [Source:HGNC Symbol;Acc:HGNC:22954] | 9794 | -0.301 | -0.0782 | No |
| 153 | FUT3 | fucosyltransferase 3 (Lewis blood group) [Source:HGNC Symbol;Acc:HGNC:4014] | 9890 | -0.311 | -0.0807 | No |
| 154 | ST8SIA3 | "ST8 alpha-N-acetyl-neuraminide alpha-2,8-sialyltransferase 3 [Source:HGNC Symbol;Acc:HGNC:14269]" | 10046 | -0.326 | -0.0876 | No |
| 155 | PLOD2 | "procollagen-lysine,2-oxoglutarate 5-dioxygenase 2 [Source:HGNC Symbol;Acc:HGNC:9082]" | 10086 | -0.330 | -0.0853 | No |
| 156 | EOGT | EGF domain specific O-linked N-acetylglucosamine transferase [Source:HGNC Symbol;Acc:HGNC:28526] | 10174 | -0.338 | -0.0866 | No |
| 157 | GBE1 | "1,4-alpha-glucan branching enzyme 1 [Source:HGNC Symbol;Acc:HGNC:4180]" | 10356 | -0.357 | -0.0951 | No |
| 158 | GALNT12 | polypeptide N-acetylgalactosaminyltransferase 12 [Source:HGNC Symbol;Acc:HGNC:19877] | 10414 | -0.362 | -0.0937 | No |
| 159 | B4GALT2 | "beta-1,4-galactosyltransferase 2 [Source:HGNC Symbol;Acc:HGNC:925]" | 10490 | -0.372 | -0.0935 | No |
| 160 | ST3GAL4 | "ST3 beta-galactoside alpha-2,3-sialyltransferase 4 [Source:HGNC Symbol;Acc:HGNC:10864]" | 10688 | -0.391 | -0.1026 | No |
| 161 | PARP11 | poly(ADP-ribose) polymerase family member 11 [Source:HGNC Symbol;Acc:HGNC:1186] | 10766 | -0.401 | -0.1022 | No |
| 162 | ART4 | ADP-ribosyltransferase 4 (inactive) (Dombrock blood group) [Source:HGNC Symbol;Acc:HGNC:726] | 10866 | -0.412 | -0.1033 | No |
| 163 | HAS2 | hyaluronan synthase 2 [Source:HGNC Symbol;Acc:HGNC:4819] | 10980 | -0.428 | -0.1052 | No |
| 164 | GALNT3 | polypeptide N-acetylgalactosaminyltransferase 3 [Source:HGNC Symbol;Acc:HGNC:4125] | 11062 | -0.440 | -0.1044 | No |
| 165 | FUT1 | fucosyltransferase 1 (H blood group) [Source:HGNC Symbol;Acc:HGNC:4012] | 11086 | -0.442 | -0.0991 | No |
| 166 | ST6GALNAC4 | "ST6 N-acetylgalactosaminide alpha-2,6-sialyltransferase 4 [Source:HGNC Symbol;Acc:HGNC:17846]" | 11164 | -0.452 | -0.0978 | No |
| 167 | QTRT2 | queuine tRNA-ribosyltransferase accessory subunit 2 [Source:HGNC Symbol;Acc:HGNC:25771] | 11420 | -0.489 | -0.1099 | No |
| 168 | FUT2 | fucosyltransferase 2 [Source:HGNC Symbol;Acc:HGNC:4013] | 11459 | -0.497 | -0.1048 | No |
| 169 | B4GALT1 | "beta-1,4-galactosyltransferase 1 [Source:HGNC Symbol;Acc:HGNC:924]" | 11530 | -0.505 | -0.1021 | No |
| 170 | PPAT | phosphoribosyl pyrophosphate amidotransferase [Source:HGNC Symbol;Acc:HGNC:9238] | 11534 | -0.505 | -0.0941 | No |
| 171 | A4GNT | "alpha-1,4-N-acetylglucosaminyltransferase [Source:HGNC Symbol;Acc:HGNC:17968]" | 11568 | -0.510 | -0.0884 | No |
| 172 | CHSY1 | chondroitin sulfate synthase 1 [Source:HGNC Symbol;Acc:HGNC:17198] | 11628 | -0.521 | -0.0846 | No |
| 173 | ST3GAL2 | "ST3 beta-galactoside alpha-2,3-sialyltransferase 2 [Source:HGNC Symbol;Acc:HGNC:10863]" | 11721 | -0.536 | -0.0831 | No |
| 174 | POFUT2 | protein O-fucosyltransferase 2 [Source:HGNC Symbol;Acc:HGNC:14683] | 11725 | -0.537 | -0.0746 | No |
| 175 | B4GALT3 | "beta-1,4-galactosyltransferase 3 [Source:HGNC Symbol;Acc:HGNC:926]" | 11748 | -0.540 | -0.0676 | No |
| 176 | B3GNTL1 | "UDP-GlcNAc:betaGal beta-1,3-N-acetylglucosaminyltransferase like 1 [Source:HGNC Symbol;Acc:HGNC:21727]" | 11943 | -0.581 | -0.0734 | No |
| 177 | UPP1 | uridine phosphorylase 1 [Source:HGNC Symbol;Acc:HGNC:12576] | 12013 | -0.595 | -0.0692 | No |
| 178 | FUT6 | fucosyltransferase 6 [Source:HGNC Symbol;Acc:HGNC:4017] | 12067 | -0.612 | -0.0634 | No |
| 179 | PARP6 | poly(ADP-ribose) polymerase family member 6 [Source:HGNC Symbol;Acc:HGNC:26921] | 12090 | -0.618 | -0.0551 | No |
| 180 | ST3GAL1 | "ST3 beta-galactoside alpha-2,3-sialyltransferase 1 [Source:HGNC Symbol;Acc:HGNC:10862]" | 12092 | -0.619 | -0.0451 | No |
| 181 | UGT2B15 | UDP glucuronosyltransferase family 2 member B15 [Source:HGNC Symbol;Acc:HGNC:12546] | 12225 | -0.652 | -0.0449 | No |
| 182 | NAMPT | nicotinamide phosphoribosyltransferase [Source:HGNC Symbol;Acc:HGNC:30092] | 12292 | -0.674 | -0.0391 | No |
| 183 | OGT | O-linked N-acetylglucosamine (GlcNAc) transferase [Source:HGNC Symbol;Acc:HGNC:8127] | 12440 | -0.721 | -0.0390 | No |
| 184 | EXTL3 | exostosin like glycosyltransferase 3 [Source:HGNC Symbol;Acc:HGNC:3518] | 12442 | -0.722 | -0.0273 | No |
| 185 | UGCG | UDP-glucose ceramide glucosyltransferase [Source:HGNC Symbol;Acc:HGNC:12524] | 12532 | -0.763 | -0.0219 | No |
| 186 | HAS1 | hyaluronan synthase 1 [Source:HGNC Symbol;Acc:HGNC:4818] | 12655 | -0.831 | -0.0180 | No |
| 187 | TIPARP | TCDD inducible poly(ADP-ribose) polymerase [Source:HGNC Symbol;Acc:HGNC:23696] | 12789 | -0.950 | -0.0131 | No |
| 188 | TNKS2 | tankyrase 2 [Source:HGNC Symbol;Acc:HGNC:15677] | 12895 | -1.336 | 0.0004 | No |
Table: GSEA details [plain text format]

  

Fig 2: GOMF\_GLYCOSYLTRANSFERASE\_ACTIVITY      
 Blue-Pink O' Gram in the Space of the Analyzed GeneSet

  

Fig 3: GOMF\_GLYCOSYLTRANSFERASE\_ACTIVITY: Random ES distribution      
 Gene set null distribution of ES for **GOMF\_GLYCOSYLTRANSFERASE\_ACTIVITY**
